# Nuclear P38: Roles in Physiological and Pathological Processes and Regulation of Nuclear Translocation

**DOI:** 10.3390/ijms21176102

**Published:** 2020-08-24

**Authors:** Galia Maik-Rachline, Lucia Lifshits, Rony Seger

**Affiliations:** Department of Biological Regulation, Weizmann Institute of Science, Rehovot 7610001, Israel; galia.maik-rachline@weizmann.ac.il (G.M.-R.); Lucia.lifshits@weizmann.ac.il (L.L.)

**Keywords:** p38MAPK, nuclear translocation, β-like importins, inflammation, cancer

## Abstract

The p38 mitogen-activated protein kinase (p38MAPK, termed here p38) cascade is a central signaling pathway that transmits stress and other signals to various intracellular targets in the cytoplasm and nucleus. More than 150 substrates of p38α/β have been identified, and this number is likely to increase. The phosphorylation of these substrates initiates or regulates a large number of cellular processes including transcription, translation, RNA processing and cell cycle progression, as well as degradation and the nuclear translocation of various proteins. Being such a central signaling cascade, its dysregulation is associated with many pathologies, particularly inflammation and cancer. One of the hallmarks of p38α/β signaling is its stimulated nuclear translocation, which occurs shortly after extracellular stimulation. Although p38α/β do not contain nuclear localization or nuclear export signals, they rapidly and robustly translocate to the nucleus, and they are exported back to the cytoplasm within minutes to hours. Here, we describe the physiological and pathological roles of p38α/β phosphorylation, concentrating mainly on the ill-reviewed regulation of p38α/β substrate degradation and nuclear translocation. In addition, we provide information on the p38α/β ’s substrates, concentrating mainly on the nuclear targets and their role in p38α/β functions. Finally, we also provide information on the mechanisms of nuclear p38α/β translocation and its use as a therapeutic target for p38α/β-dependent diseases.

## 1. Introduction

The p38 mitogen-activated protein kinase (p38MAPK, termed here p38) is a signaling protein kinase that operates within a signaling cascade to transmit extracellular signals to their intracellular targets. The p38 cascade is one of four similar cascades that are all key communication lines between the plasma membranes and the nucleus, and thereby, it is involved in fundamental cellular processes, including stress response, proliferation, differentiation and others [1,2,3]. The four MAPK cascades are extracellular signal-regulated kinase (ERK) 1/2 [4], c-Jun N-terminal kinase (JNK [5]), p38 [6], and ERK5 [7]. The MAPK cascades transmit signals via a sequential activation of protein kinases, which are organized in 3–5 tiers (MAP4K, MAP3K, MAPKK, MAPK, and MAPK activated protein kinases (MAPKAPKs also termed MKs)). Each of these tiers includes more than one kinase (e.g., 4 isoforms at the p38 tier), and the components involved, while the number of tiers may vary between cell lines or under different conditions (see the scheme of the MAPK cascades in ref [8]). In this review, we focus on p38 [9,10], whose cascade is composed of many kinases at the MAP4K and MAP3K levels, MKK3/6, and perhaps MKK4 at the MAPKK tier, p38α−δ at the MAPK tier, and several MKs at the next tier (MNK1/2, MSK1/2, MK2/3, and MK5). Interestingly, unlike the other MAPKs, p38 can also be activated via MKK-independent pathways, either by ZAP/LCK-mediated Tyr phosphorylation [11] or by interaction with TAB1 [12]. The downregulation/inactivation of the p38 cascade is regulated by various phosphatases, among them are several dual specificity phosphatases termed MAPK phosphatases (MKPs) that operate directly on the MAPKs [13]. As in all MAPK cascades, p38 transmits signals initiated by various agents, including cytokines and environmental queues, but it is known to operate mainly as a mediator of stress responses. Thus, the kinase is a key regulator of metabolic, oxidative, and endoplasmic reticulum (ER) stresses, but it plays an important role in other physiological processes such as cell cycle, senescence, differentiation, and several aspects of immunological processes.

Being responsible for the various distinct and even opposing fundamental cellular processes, the p38 cascade needs to be tightly regulated. Indeed, several regulatory mechanisms that determine the specificity of the cascade have been identified, including the duration and strength of the signals [13,14], which are controlled mainly by dual specificity phosphatases [15,16], scaffold proteins [17], and dynamic subcellular localization of the cascade’s components [18]. Importantly, the central roles of the cascade suggest that its dysregulation may cause various diseases. Indeed, p38 was shown to participate in the induction of pathologies such as inflammation-related diseases [19], autoimmune diseases [20], some types of cancer [6], and other pathologies, as specified later in this review. Interestingly, unlike other MAPKs, p38 demonstrates distinct and even opposing effects in different cancers, as it was shown to serve either as a tumor suppressor [21] or tumor promoter [22]. It was also shown that in some cases, it can perform both activities in different stages of cancer development [23]. Although all p38 isoforms have been implicated in the processes listed above, they can be divided into two somewhat distinct subgroups: p38α and p38β (p38α/β) versus p38γ and p38δ. In this review, we focus on p38α/β, mainly discussing the physiological and pathological roles of these protein kinases, providing information on nuclear p38α/βs and their substrates as well as the importance of their phosphorylation specificity. We also describe the mechanisms involved in the nuclear translocation of p38α/β and compare it to other mechanisms of nuclear shuttling. The fact that p38α/β has so many nuclear targets indicates that the prevention of their nuclear translocation may affect their physiological and pathological functions. Indeed, we show that prevention of the nuclear translocation can be used as a tool to combat inflammation and cancer.

## 2. Physiological Roles of Nuclear p38α/β

The p38α/β are best known for their involvement in stress signaling, and indeed, these kinases as well as JNKs were initially termed stress-activated protein kinases (SAPKs [24]). However, it was established that their activity is not confined to stress responses, and under some conditions, the p38α/β may participate in the regulation of other processes, such as proliferation, differentiation, immune response, migration, and apoptosis. In many cases, p38α/β mediate their effects by activating and regulating transcription factors. One interesting example is the modulation of endoplasmic reticulum (ER) stress in breast cancer cells, which is mediated by the p38α/β-dependent activation of the transcription factor XBP-1 that decreases the expression of the ER protein ERp29 [25]. Another example is the transcriptional inhibition of autophagy genes downstream of p38α/β in response to oxidative stress in HeLa cells [26]. Other stresses such as UV radiation translational inhibition and others were shown to operate by p38α/β−activated transcription factors, such as ATF, MEF2, Elk1, and p53 [3]. However, p38α/β can also affect other regulators (e.g., MKs, proteasome, EGFR [27]) to coordinate their signaling. Notable transcription factor-independent targets that exert p38α/β functions are cell cycle regulators that modulate (usually inhibiting) cell cycle progression. Thus, various stresses induce the downregulation of cyclinD, thereby arresting cells at G1 [28]. In addition, p38α/β cascades were shown to induce the expression of CDK inhibitors, activate p53, or inhibit the transcription factor E2F and the G2/M regulator Cdc25B phosphatase, all leading to the inhibition of cell cycle progression [6,29]. By contrast, in some systems, p38α/β seem to enhance proliferation. For example, such effects were detected in hematopoietic cells and in some cancer cell lines [30]. These differential effects may be mediated by changes in the duration of p38α/β signals, where transient signals lead to fibroblasts’ proliferation, while sustained signals induce cell cycle arrest [31]. However, the molecular mechanisms by which p38α/β are involved in proliferation have not been fully deciphered yet. Thus, the effects of p38α/β on the regulation of stress response or cell cycle progression are well-reviewed (e.g., [6,27,32,33]), and we will not elaborate on these effects. However, not less important are the roles of p38α/β in regulating protein degradation and the translocation of proteins upon stimulation. The molecular mechanisms involved are described in detail next.

### 2.1. 38α/β Regulation of Protein Degradation

The role of p38α/β in the regulation of protein degradation is widespread, mainly upon stress signals, and may involve several distinct mechanisms in both the cytoplasm and the nucleus. One such mechanism that mostly occurs in the nucleus involves phosphorylation of ubiquitin E3 ligases, such as Siah2, which is known to regulate PHD3 that further controls the stability of the transcription factor HIF1 α. p38α/β phosphorylate Siah2 on Ser24 and Thr29, thereby facilitating its activity towards degradation of PHD, and in turn destabilization of HIF1 α [34,35]. Similarly, p38α/β phosphorylate the E3 ligase Skp2 at Ser64, leading to enhanced degradation of the transcription factor Nkx3-1 and thereby blocking its effects on estrogen receptor-mediated gene expression [36]. Another mechanism involves the phosphorylation of the ubiquitination target which can either facilitate or inhibit the ubiquitination process. Examples for enhanced degradation are the phosphorylation of RBP-Jk at Thr339 which subsequently induces its degradation [37], or phosphorylation of p300 at Ser 1834 (together with AKT) that induces its degradation to allow DNA repair [38]. On the other hand, phosphorylation of the inflammation regulator TRIM9s at Ser76/80 stabilizes it, thereby causing a positive feedback loop for the degradation of the upstream MKK6 [39]. Interestingly, p38α/β may also regulate proteasomal activity and localization to govern protein stability in general. It was shown that osmotic stress inhibits proteasome by p38α/β-dependent phosphorylation of the proteasome subunit Rpn2 at Thr273, which is important for peptide degrading activity [40]. This inhibitory effect was supported by the finding that p38 inhibitors elevate proteasome activity under varying conditions [41]. In addition, p38α/β may regulate the subcellular localization of the proteasome by phosphorylating the proteasome-binding protein PI31. Consequently, this phosphorylation facilitates the association of the proteasome with the motor dynein complex, and regulates its transport on axons [42]. Other proteins whose stability is regulated by direct p38 phosphorylation are Cdt1, HBP1, p18Hamlet, Rb1, SRC3, CDC25A/B, CyclineD1/3, TACE, p53, Snail, Twist, Nav1.6, PGC1 α, HuR and Drosha [27,43]. Thus, p38α/β use various molecular mechanisms to regulate stimulation-dependent proteins stability.

### 2.2. p38α/β Regulation of Stimulated Nuclear Translocation

Another important process that is regulated by p38α/β is the dynamic change of protein localization upon stimulation. As described above regarding the regulation of protein degradation, the effect of p38α/β on nuclear translocation can be either global or specific to certain phosphorylated proteins. The global effects may be derived by p38α/β phosphorylation of either nuclear pore proteins or of karyopherins (importins/exportins). Indeed, it was shown that the nuclear pore proteins Nup62, Nup153, and Nup214 are phosphorylated by p38 (or ERK), and this phosphorylation inhibits the global nuclear protein shuttling initiated by viruses that affect the heart such as the encephalomyocarditis virus [44]. A similar effect was detected in cardiomyocytes of failing hearts in rats and humans, where p38α/β phosphorylation mediates the rearrangement of nuclear pores, leading to a decreased uptake of nuclear localization signal (NLS)-containing proteins [45]. As for karyopherins, it was shown that p38α/β regulate the expression of the beta-like importins (Imp) Imp7 and Imp8 [46], which are important for the nuclear translocation of various signaling proteins. Aside from the global changes, p38α/β is known to phosphorylate the translocating proteins themselves to mediate either nuclear accumulation or nuclear export. For example, the active SMAD3 phosphorylation by p38α/β upon TGF β stimulation reduces the rate of its nuclear translocation [47]. A similar effect was detected for FOXO3 α, which is phosphorylated by p38α/β at Ser7 to promote its nuclear localization [48]. The nuclear translocation of RhoA due to p38 phosphorylation upon LPS treatment and of actin upon TPA stimulation [49] was reported as well [50]. On the other hand, p38α/β phosphorylation may be responsible for the nuclear export of proteins; the most famous among them are its downstream MKs. It was shown that MK2/3 contain an NLS, which directs them to the nucleus of resting cells. Following phosphorylation by p38α/β, MK2/3 are exported to the cytoplasm, due to unmasking of the C-terminal NES of the MK2/3 (reviewed in [51]). Moreover, MK5 contains an NLS as well, and can be found in the nucleus under certain conditions. However, its export after p38α/β phosphorylation seems to be mediated not only by exposure of NES but also by anchoring to ERK3/4 [52]. Other proteins whose localization is directly regulated by p38α/β phosphorylation are retinoic acid receptor-γ, [53], androgen receptor [54], estrogen receptor-α [55], 5-lipoxygenase [56], the Hippo pathway transcription factor TEAD4 [57], as well as other proteins (NFATc4, Xbp1s, Drosha, CRTC2, HuR, Rabenosyn5, Lamin-B, FGFR1, PIP4K2B, EZH2, and Tripeptidyl-Peptidase II) as specified in previous reviews [27,43]. Thus, p38α/β use several distinct mechanisms for the regulation of nuclear translocation of proteins upon various stimulations.

## 3. Role of Nuclear p38α/β in Pathologies

Abnormal activity and dysregulation of the p38α/β cascade are associated with a variety of diseases. Indeed, p38α/β were implicated in the induction and maintenance of several pathologies such as inflammation [19], cancer [6], and autoimmune diseases [20] mentioned above, but also Friedreich’s ataxia [58], Parkinson’s disease [59], Alzheimer’s disease [60], cardiac hypertrophy [61], hypoxic nephropathy [62], and diabetes [63]. In many cases, the role of p38α/β is not direct, but it is mediated by p38α/β-regulated inflammation, which in turn contributes to the development of the diseases. For example, Parkinson’s disease is induced in part by neuroinflammation associated with glial cells [61], and colorectal cancer often develops due to initial inflammatory disease of the colon [64]. Moreover, p38α was first identified due to its involvement in the production of pro-inflammatory cytokines upon endotoxin treatment, mainly via nuclear processes [65]. It was later found that p38α/β are involved in the production of pro-inflammatory cytokines, such as TNF-α, IL-1 β, IL-2 IL-6, IL-7, and IL-8, and also in the regulation of other inflammation mediators such as Cox2 [66,67]. Although many of these effects involve nuclear processes in some instances, it may be regulated also by translation, due to AU-rich elements (ARE) in the 3′ untranslated region of their mRNA. The presence of these elements is known to shorten the half-life of mRNA containing them or block their translation, mainly due to the phosphorylation of ARE binding proteins such as HuR [68] by MK2 downstream of p38α/β) [69]. The pro-inflammatory cytokines are known players in many inflammation-related diseases such as inflammatory bowel diseases (IBD), psoriasis asthma, rheumatoid arthritis, inflammation-induced cancer, and more [66]. However, the actual trigger for inflammation in some systems, either acute or chronic, is not known, but it still requires p38α/β for its mediation. The means by which p38α/β are involved in these processes and the upstream components involved need further investigation. Due to the involvement of p38α/β in several inflammatory diseases, it became clear that specific inhibitors of these kinases should become a beneficial therapeutic approach. Indeed, in the past decade, more than 20 specific inhibitors of p38α/β were developed and proven very effective in pre-clinical investigations, demonstrating good tolerability and efficacy in several mouse models [70,71]. However, when subjected to clinical trials, the effects were much less favorable. Apparently, except for one inhibitor, pirfenidone, which by itself demonstrated a weak and unselective effect, no durable therapeutic effects have been detected for any of the others tested. The reasons for these failures among the different drugs are numerous, and while in some cases they were toxic, the more common problem was that after an initial good response, there was a rebound effect that increased inflammation within weeks. The reason for this rebound is still not clear and is currently under investigation.

In the past decade, substantial research has been devoted to studying the role of p38α/β in cancer, confirming that these kinases can act either as tumor promotors, or more frequently, as tumor suppressors [6,30]. The tumor suppressor effects were corroborated by immortalized MKK3/6 knockout fibroblasts that were shown to have a higher tendency to develop xenografts in nude mice [72]. Moreover, it was shown that the expression of MKK3/6, or other components of the cascade, is reduced in many cancers [73]. The function of p38α/β as tumor suppressors usually affect early stages of tumor initiation, and it generally involves either an inhibition of cell cycle progression, enhanced apoptosis, senescence, or differentiation. Similar to the effects in non-transformed cells, the inhibition of cell cycle by p38α/β in tumors can be mediated by the direct or indirect phosphorylation of several nuclear substrates. Among them are CyclinD, whose inhibition may cause apoptosis in colorectal cancer cells [28], p53, that leads to the upregulation of p21Cip1/WAF1, GADD45, and 14-3-3 proteins to cause cell cycle arrest [74], RB1, which prevents the metastasis of prostate cancer [75], and others [6]. Additionally, it was shown that p38α/β facilitate the production of apoptotic cytokines such as TNF-α [76]. The terminal differentiation of cancer was detected in rhabdomyosarcoma cells overexpressing MKK3 or MKK6 [77], and premature senescence was related to p38α/β-induced phosphorylation of the transcription factor HBP1 [78]. Some other effects on tumor promotion, although less frequent, may be mediated via inflammation in relevant cancers. Other mechanisms that may initiate cancer by p38α/β are elevated migration/invasion, increased angiogenesis [6], or a direct effect on proliferation [79,80]. As mentioned above, p38α/β are central regulators of inflammation, which in many cases is involved in cancer initiation and progression [81]. Indeed, some of the specific p38α/β inhibitors that have been developed over the years, although having failed in inflammation-related clinical trials, were proven useful in treating cancers [82]. Interestingly, in some cancer cells, p38α/β may facilitate migration by several mechanisms, including an enhanced production of chemoattractants [83] or reduced expression of fibulin 3, which is a cell migration blocker [84]. Finally, the involvement of p38α/β in angiogenesis, which supplies blood vessels to the tumors and enhances their growth, was shown to occur in head and neck cancer [85]. Some reports have shown that the molecular mechanisms involved include expression of vascular endothelial growth factor (VEGFA) and hypoxia-inducible factor 1 α (HIF1 α [30]. Interestingly, in models of colorectal cancer, p38α/β may act as either tumor suppressors or promoters in different stages of cancer development [23].

## 4. p38α/β’s Substrates and their Phosphorylation Specificity

More than 150 direct substrates of p38α/β have been identified so far [27,43,86,87,88,89]. However, since their minimal consensus phosphorylation sequence on their substrates (Ser/Thr-Pro) is so limited, and estimated to be present on the surface of approximately 50% of the cellular proteins, the actual number is likely to increase. Moreover, the effects of p38α/β are propagated by their MKs, and therefore, the number of phosphorylated proteins downstream of p38α/β may reach several thousands. Direct substrates of p38α/β were categorized into several subgroups in a previous review [43], including DNA binding proteins, RNA binding proteins, Ser/Thr kinases, regulatory proteins, as well as membranal, endosomal, and structural proteins. Additional information has been accumulated over the years on the role of p38α/β in activating their downstream protein kinases (MKs), transcription factors, and other regulatory elements [6,9]. The phosphorylation of these substrates is important for orchestrating the various cellular processes that may be, under certain circumstances, opposing signals. For example, the stress-related transcription factors ATF2, MEF2s, and CHOP have long been known to transmit p38α/β stress signals, while Elk1 and cFos may transmit its downstream mitogenic signals [3]. These distinct effects raise the question as to how the specificity of p38 signals is regulated.

As all other MAPKs, activated p38α/β execute their functions through the phosphorylation of downstream proteins. To the best of our knowledge, unlike ERK [90], no phosphorylation-independent effects of p38α/β have been identified. The full (Pro-Xaa-Ser/Thr-Pro) or minimal (Ser/Thr-Pro) consensus phosphorylation sites of all MAPKs are similar to each other [91]. Therefore, the interaction with the residues in the phosphorylation site is not sufficient to provide p38α/β specificity to its substrates. Rather, the specificity is mostly achieved by docking motifs that are localized on the substrates (D, DEF) that interact with specific docking sites on p38α/β (CD, Hydrophobic pocket). Thus, a docking motif that is found in many substrates of p38α/β is a D domain with consensus sequence Arg/Lys_2_-Xaa_2-6_-Φaa-Xaa-Φaa (where Φaa is a hydrophobic residue). The D-domains in the substrates bind to their counterpart docking site on p38α/β termed common docking motif (CD), which is composed of three negatively charged residues and at least two hydrophobic residues [92]. The CD domain and the catalytic site of p38α/β are located in distinct regions of the kinases, which allows the phosphorylation to occur at a fixed distance from the substrates D domain [93]. Binding of the D domain to the CD occurs in many cases when p38α/β are inactive, indicating that it may be responsible for a pre-activation association, which facilitates the rates of phosphorylation. Interestingly, similar to the consensus phosphorylation sites, also the D–CD domains interactions are nearly identical among all MAPKs, and therefore, p38α/β specificity requires extra determinants. Indeed, it was shown that in some cases, p38, ERK, and JNK may interact with distinct residues within the D-domain [94], and that two hydrophobic residues in the domain may determine specificity to some extent [95].

However, the differences between the hydrophobic residues cannot fully explain the specificity of p38α/β phosphorylation. Rather, this is likely explained by a second docking site on p38α/β substrates termed DEF (docking site for ERK, also known as FXF), which consists of two Phe residues separated by one residue and is often followed by a Pro residue [96]. The DEF domain binds the hydrophobic pocket in p38α/β, which is located at several residues C-terminal of the activatory Thr-Gly-Tyr phosphorylation site of the kinases. Structural studies show that the hydrophobic pocket is formed only upon phosphorylation, and therefore, unlike the interaction of the D-domain, only active p38α/β molecules bind to the DEF motif. Finally, it was also shown that p38, but not p38α, possesses intrinsic autophosphorylation activity, which may by itself elevate the basal activity of the kinase [97]. Thus, the combination of the three substrate interaction motifs together with intrinsic kinase activity contribute to the substrate specificity and affinity required for the proper p38α/β’s functions under various conditions. In addition, other regulators that affect signaling specificity may contribute to the kinase activity, including the level of substrates’ expression and their stability after stimulation as well as compartmentalization, scaffold proteins, or distinct phosphatases in a given cell. These regulating elements may also lead to sustained rather than transient activation of p38α/β, which is another specificity determinant. This is because the sustained phosphorylation results in the substrate’s phosphorylation at later stages after stimulation [98], as seen in cases of cell cycle facilitation versus senescence [31,99]. Taken together, these effects regulate the outcome of the p38α/β signal that are important both for the physiological and pathological fates of the cells.

## 5. Subcellular Localization of p38α/β and their Substrates

One of the mechanisms that determines the ability of MAPKs to phosphorylate distinct substrates upon varying conditions or cell lines is the cellular localization of the substrates’ phosphorylation [100]. Indeed, the large number of p38α/β’s substrates was shown to localize in various distinct compartments, including the nucleus, cytoplasm, cytoskeletal elements, and other sites [86]. In many cases, the distribution of the substrates is dynamic and changed upon stimulation, or in different cell lines. As mentioned above, some of these changes can be regulated by p38α/β, but others are regulated by other signaling proteins. The dynamic changes in localization raise the question of where the phosphorylation by p38α/β is actually taking place. Unexpectedly, the transcription factors that serve as substrates and function within the nucleus are almost always cytoplasmic in resting cells and translocate to the nucleus upon stimulation. As mentioned above, the translocations of some of these transcription factors are regulated by p38α/β. For example, the transcription factor ATF2 is phosphorylated by p38α/β to facilitate its dimerization with other AP-1 transcription factors, which enhances nuclear translocation [101]. Additionally, p38α/β phosphorylate the transcription factor Xbp1s at Thr48 and Ser61 enhancing migration to the nucleus, thus regulating glucose homeostasis in obesity [102]. The nuclear translocation of the transcription factor MEF2A is regulated by p38α/β as well [103], leading to the expression of neonatal myosin heavy chain in C2C12 myoblasts. Thus, counterintuitively, the phosphorylation of most, if not all, transcription factors by p38α/β may take place primarily in the cytoplasm, although some phosphorylations can also occur in the nucleus upon translocation. Similar effects were shown for other effectors of p38α/β, which translocate to the nucleus upon stimulation. Importantly, the localization of some substrates might be cell-type or condition-dependent [86], but others are less variable, and they are either constantly localized in the cytoplasm (e.g., keratin-8), in the nucleus (e.g., histones), or in the nucleus of resting cells followed by export upon stimulation (e.g., MKs). A list of various p38α/β substrates, which includes several transcription factors but mostly other proteins (Table 1), indicates that about 30% of the p38α/β substrates are confined to the nucleus under most/all conditions, and therefore, their phosphorylation upon stimulation should be nuclear. Almost all these proteins were shown to participate in the regulation of cancer and inflammation at least under some conditions. These effects are supported by our recent findings that the inhibition of nuclear p38α/β translocation prevents DSS-induced colitis and DSS/AOM-induced colon cancer [80], confirming the importance of nuclear p38α/β. However, it should be noted that the involvement of nuclear p38α/β in cancer and inflammation was not always confirmed, and the effects are sometimes cell line-dependent. Taken together, the information included in Table 1, as well as our recent study, clearly indicate that p38α/β are involved in either positive or negative regulations of cancer and inflammation.

The ability of p38α/β to phosphorylate substrates in the cytoplasm as well as in the nucleus and the role of nuclear p38α/β in the regulation of cancer and inflammation diverted attention toward the subcellular localization of these kinases. Thus, it was initially shown that p38α/β may be localized in the cytoplasm of resting cells, and similarly to ERK [104], it may translocate to the nucleus upon stimulation [105,106]. In that case, the kinases remain in the nucleus for minutes to hours, after which they are exported back to the cytoplasm. Surprisingly, in other systems, p38α/β were detected in the nucleus of resting cells [107] and were exported out of the nucleus shortly upon stimulation [108]. The nuclear localization in resting cells likely occurs because of continued stress signals in these cells, or due to the expression of specific nuclear anchors that attract p38α/β to the nucleus. However, this localization seems to occur in only a limited number of systems, whereas, in most cases, p38α/β are localized in the cytoplasm of resting cells. As for the mechanisms that regulate the dynamic subcellular localization, similarly to ERKs [109], it was shown that the cytoplasmic localization of p38α/β is mediated by various cytoplasmic anchoring proteins such as PTP-SL [106], keratins [110], and others [111]. Upon stimulation, the p38α/β detach from their anchors by mechanisms that may [112] or may not [113] require the activating TGY phosphorylation. Then, p38α/β translocate to the nucleus via the nuclear pore and can stay there for minutes to hours, either in the nucleoplasm, bound to chromatin [114], or nuclear proteins [108]. Interestingly, the mechanism of export either in cells with constant nuclear p38α/β, or in later stages upon stimulation-induced translocation, involves binding to the nuclear export signal (NES)-containing p38α/β’s substrate MK2 but probably not to those containing MK3 and MK5 [108,112].

## 6. Mechanism of Nuclear p38α/β Translocation and the Effect of its Inhibition

**Figure 1:**
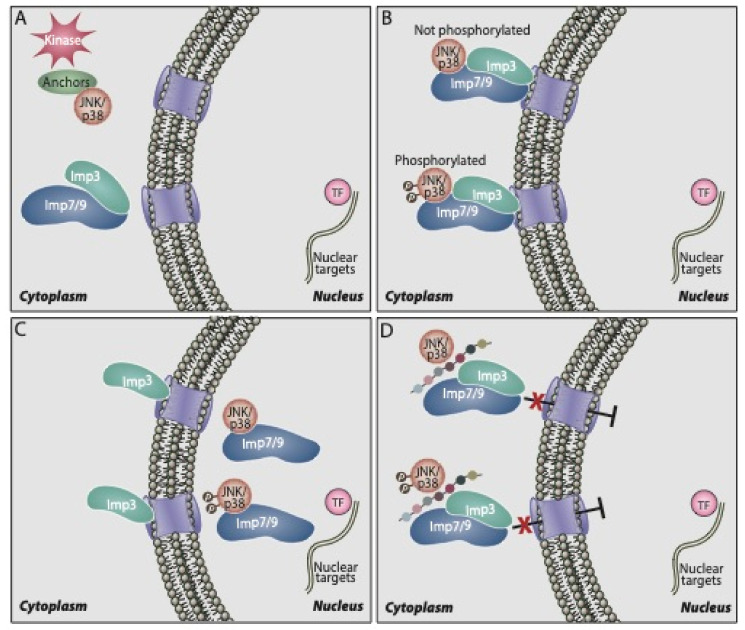
Scheme showing the mechanism of nuclear p38α/β translocation and its inhibition by the PERY peptide. **A**. p38α/β are localized in the cytoplasm of resting cells and some of the molecules are phosphorylated upon stimulation. **B**. Phosphorylated or non-phosphorylated p38α/β bind to a dimer of Imp7/3 or Imp9/3, which escort them to the nuclear pores. **C**. Imp3 stays outside, while Imp7 or Imp9 escort the p38α/β through the nuclear pores, to the nucleus, where they dissociate from the importins, and are free to phosphorylate their substrates. **D**. Translocation of the kinases to the nucleus can be inhibited using the inhibitory PERY peptide that was synthesized according to the sequence of the p38α–Imp binding site as described in the text.

Although it is clear that p38α/β shuttle to the nucleus, either upon or without stimulation, the mechanism that regulates this is not fully understood. Similarly to ERK and JNK, p38α/β do not contain the canonical nuclear localization signals (NLS). In addition, they do not seem to interact with the classical Impα/β [111], or use passive diffusion for their nuclear shuttling. On the other hand, our group has recently shown that the nuclear translocation is mediated by three β-like importins, Imp3, 7, and 9 [115]. Thus, we found that upon stimulation, p38α/β as well as JNK1/2 are released from their cytoplasmic anchoring proteins and interact with either Imp7 or Imp9, each one in a complex with Imp3 (see the schematic representation in Figure 1). Then, the trimers formed (Imp7/Imp3/kinase or Imp9/Imp3/kinase) are shuttled to the nuclear pore, where Imp3 remains, while Imp7 or Imp9 escort the shuttling p38α/β or JNK1/2 into the nucleus [111]. In the nucleus, the small GTPase Ran dissociates the importins from p38α/β, and the latter are freed to execute their functions. This mechanism has some similarity to the translocation of ERK, which is detached from its anchoring protein upon stimulation and interacts with Imp7 that escorts it to the nucleus [18,116,117]. The Imp7 binding site of p38α/β resides in the N terminus of the kinase, and it is composed of at least nine residues: PERYQNLSP. Indeed, deletion of this region or substitution of its residues to Ala residues prevents the interaction [80]. Interestingly, the nuclear translocation of p38α/β may require HSP70 interaction in the nucleus [118], rely on microtubules and dynein, which may indicate that the translocation is helped by a trafficking machinery [112] and might involve SUMOylation of the kinases [119]. However, the mechanisms by which these components participate in the translocation and how they are related to the importins involved are not clear.

In previous studies on the nuclear translocation of ERK, our group designed a myristoylated peptide (EPE peptide) based on the ERK interaction site with Imp7 [120,121]. When added to cells, the peptide completely abolished the interaction between ERK and Imp7, and it prevented both stimulated and non-stimulated translocation of the kinase, indicating that the great majority of the translocation is mediated by Imp7, but not by passive diffusion [122] under both conditions. The potency of the peptide varied between cell lines, completely abolishing the proliferation of melanoma and other ERK-addicted cancer cell lines, but not the growth of non-transformed cells [120,123]. The peptide also diminished the growth of melanoma xenografts, better than the Raf inhibitor vemurafenib. Based on the successful development of the EPE peptide, we developed a myristoylated peptide targeting the binding site of p38α/β to Imp7/9, which we termed the PERY peptide. As expected [80], the peptide completely abolished the interaction of p38α/β to both Imp7 and Imp9, preventing the nuclear translocation of the kinases and the phosphorylation of nuclear targets. Since p38α/β signaling is involved in the proliferation of only limited types of cancers [6], the PERY peptide inhibited the proliferation of some breast cancer and melanoma cells but not of other cancers. In most cases, the effects were similar to those of the commercial p38 activity inhibitors, indicating that the effect on inflammation and cancer is mediated mainly by the nuclear p38α/β. Importantly, the peptide inhibited DSS-induced colon inflammation (colitis-like) and impressively, it demonstrated a significant inhibitory effect on a model of colitis-associated colon cancer [80]. Its effect was stronger when added throughout the DSS treatment than when added during the last two cycles of the treatment, and it was much stronger when compared to the effect of the commercial p38 activity inhibitor. Moreover, we have shown that the effect is mediated by macrophages [80] and not via enterocytes or other colon cells. Hence, these results clearly indicate that the nuclear translocation plays a role in the induction of inflammation and not the proliferation of colon cells. Taken together, we demonstrated that the nuclear translocation of p38α/β (and JNK) is important for the induction of inflammation, which may further lead to the development of some cancers. Additionally, it can be involved in the induction of proliferation of other cancer types such as triple negative breast cancers. Moreover, the results demonstrate that inhibiting the nuclear translocation of p38α/β may serve as a therapeutic strategy to combat various cancers as well as inflammatory diseases (Table 1).

**Table 1:** Nuclear substrates of p38α/β and their role in cancer and inflammation. More than 120 substrates of p38α/β were found in several reviews [27,43,86,87,88,89], and the translocation of each one of them was inspected in various databases. Substrates with constant (> 80%) nuclear localization in all cell lines described are shown under “Mostly Nuclear Proteins”, while proteins that are mostly nuclear in resting cells but are exported to the cytoplasm after stimulation are shown under “Nuclear Export”. The role of the p38α/β phosphorylation, as well as their general involvement in cancer or inflammation (independent of the phosphorylation in the nucleus) is described for each nuclear substrate. ND—not determined.

Localization	p38-phosphorylated Protein	Role of Phosphorylation	Involvement in Cancer	Involvement in Inflammation
	Cyclin D3	Targets cyclin D3 for proteasomal degradation [124].	Together with CDK6 regulates cell metabolism to promote cancer [125].	Together with CDK6 phosphorylates NFκB to induce inflammatory gene expression [126].
**Mostly Nuclear Proteins**	E47	Promotes MyoD/E47 association and muscle-gene transcription [127].	Induces EMT and therefore may facilitate tumor formation [128].	Required for the efficient recruitment of GR (anti-inflammatory) to chromatin [129].
	FBP2 (KSRP)	Controls stability of myogenic transcripts [130].	Regulates c-Fos RNA stability and therefore cancers [131].	Induce pro-inflammatory genes upon resveratrol treatment [132].
	FBP3	Controls prothrombin expression [133].	May regulate Myc expression [134].	May be involved in thrombin-induced inflammation [133].
	H2AX	Chromatin remodeling. Involved in G_2_ checkpoint that protects cells from DNA breaks [135].	Phosphorylation of Ser139 by RSK (the same site phosphorylated by p38) inhibits cell transformation [136].	Colonocytes from ulcerative colitis patients showed an increase in H2AX content. Not necessarily related to phosphorylation [137].
	H3	Related to chromatin remodeling and chromosome condensation [138].	p38 phosphorylation of Ser10 causes aggressive gastric cancer [139].	p38-dependent H3 phosphorylation may mark promoters for increased NFκB recruitment and inflammation [140].
	HBP1	Stabilizes the proteins that leads to cell cycle inhibition [141].	Inhibits cell cycle and functions as a tumor suppressor [78].	Promote vascular inflammation in atherogenesis [142].
	Id2	Regulates transcription, cell cycle, and differentiation [143].	Participate in VHL inactivation in cancer [144].	Maintains regulatory T cell to suppress inflammatory diseases [145].
	IWS1	Likely regulates RNA processing and export [89].	Regulates trimethylation of Histone H3 that may lead to cancer [146].	ND
	JDP2	Phosphorylation at Thr148 likely leads to proteasomal degradation (as with JNK [147]).	Implicated in progression and suppression of different cancers [148].	Involved in liver inflammation [149].
	MEF2d	Regulates recruitment of proteins to specific genes [150].	Enhances proliferation migration and invasion in pancreatic cancer [151].	Regulates IL-10 production in microglia to protect neuronal cells from inflammation-induced death [152].
	Mnk2b	Induces activation [153].	Mnk2b is oncogenic, by enhancing eIF4E phosphorylation [154].	MNK2 is involved in adipose tissue inflammation (possibly both isoforms) [155].
	MSK1	Induces activation [156].	Induces the transcription of immediate-early oncogenes [32].	Activation of the pro-inflammatory NF-κB signaling pathway through MSK1 in microglial cells [157].
	MSK2	Induces activation [158].	Induces the transcription of immediate-early oncogenes [32].	Plays a role in limiting Toll-like receptor-driven inflammation [159].
	P18Hamlet (Znhit1)	Stimulates p53-dependent apoptosis [160].	Regulates p53 and therefore cancer [160].	May affect p53-dependent inflammation [160,161].
	P53	Regulates apoptosis [162].	Tumor suppressor [161].	Suppressor of inflammation and autoimmunity [161].
	PGC-1α	Regulates cytokine-induced energy expenditure [163].	PGC-1α expression is altered in tumors and metastasis in relation to modifications in cellular metabolism [164].	Connects oxidative stress and mitochondrial metabolism with inflammatory response and metabolic syndrome [165].
	PPARalpha	Plays a role in cardiac metabolic stress response [166].	Modulates metabolic pathways and attenuates kidney tumor growth [167].	Exerts a major anti-inflammatory action in human liver [168].
	Ranbp2	Probably regulates SUMOylation and myotube formation [89].	Involved in inflammatory myofibroblastic tumor formation [169].	Inflammatory myofibroblastic tumor with RANBP2 and ALK gene rearrangement [169].
	Rb1	Mediates Fas-effects on inactivation of Rb1, independent of CDKs [170].	Functions as a tumor suppressor. Inactivation induces retinoblastoma and other cancers [171].	RB inactivation enhances pro-inflammatory signaling that can lead to cancer [172].
	RNF2	Modulates the expression of transcription factors and histone 2B acetylation [173].	Monoubiquitinates H2AK119 at the promoter of LTBP2, thus regulates TGFβ signaling to induce melanoma [174].	Inhibit interferon-dependent responses that may include inflammation [175].
	Rpn2	Negatively regulates proteasome activity [40].	Promotes metastasis of hepatocellular carcinoma [176].	Downregulated the inflammatory-associated JAK1/STAT3 pathway [177].
	RUNX2	Increases transcriptional activity [178].	Abnormally expressed in prostatecancerand associates with metastatic disease [179].	May have a role in the inflammatory remodeling of the collagen matrix [180].
	SPF45	Regulates alternative splicing site utilization [181], which may lead to multidrug resistance phenotypes [182].	The phosphorylation inhibits proliferation and therefore may block cancer [181].	Highly expressed in lung’s inflammatory cells, which might be involved in their function [182].
	SRC3	Controls the dynamics of interactions with RARalpha to facilitate gene activation [183].	Promotes breast and prostate cancer cell proliferation and survival [184].	Regulates inflammation during wound healing [185].
	AHNAK	Probably induces its differentiation-related activity [89].	Promotes metastasis through TGF-β-mediated EMT [186].	Silencing of AHNAK in dental pulp cells led to reduced inflammation-related proteins [187].
**Nuclear Export**	c/EBPalpha	Inhibits enhancer activity [188].	Suppresses tumor metastasis and growth in gastric cancer [189].	Interacts with NF-κB to regulate inflammation [190].
	c/EBPbeta	Activates enhancer activity [191].	Regulates tumor progression [192].	Induces inflammation and ER stress [193].
	ERalpha	Induces activation and nuclear export [55].	Functions as an oncogene in breast cancer [194].	Abnormal ERalpha signaling leads to inflammation [195].
	MK2	Induces activation [196].	Plays a role in the induction of lung cancer [197]. Activates cancer-related proteins (Cdc25B/C, Plk1, and TSC2) [198].	Plays a role in inflammatory pulmonary diseases [197]. Regulates inflammatory cytokines, transcript stability, and critical cellular processes [69].
	MK3	Induces activation [199].	Leads to pancreatic cancer growth [200].	Induces TNF biosynthesis and inflammation [201].
	MK5	Induces activation [202].	Induces breast cancer [203].	Phosphorylates HSP27 to induce inflammation [204].
	MRF4	Reduces transcriptional activity [205].	May regulate hairy cell leukemia (HCL) [206].	ND
	NFATc4	Activation and nuclear export [207].	Correlates with decreased proliferation and poor prognosis of ovarian cancer [208].	Involved in the secretion of inflammatory factors [209].
	NR4A	Regulates dopamine synthesis genes [210].	Has both tumor suppressor and oncogenic functions in different cells [211].	May contribute to the cellular processes that control inflammation [212].
	Pax6	Elevates transcriptional activity [213].	Induces cell proliferation in lung cancer [214].	ND

## 7. Concluding Remarks

The response of cells to stress and other extracellular stimuli leads to the activation of several signaling pathways, including primarily those of p38α/β and JNK. The p38α/β signaling cascade is well-known for its ability to transmit stress signals to various targets within the cells. Thus, it regulates the activity of many transcription factors involved in stress response, and it was shown to also regulate cell cycle, RNA processes, and cytoskeletal elements. Here, we discussed in detail its involvement in the degradation and nuclear translocation of many proteins, which is an effect that may be in both cases either global, by affecting executing enzymes, or individual by acting specifically on degrading/translocating molecules. Regarding protein degradation, the global effects may be mediated by regulating proteasome or ubiquitin ligases, while the phosphorylation of individual substrates may either stabilize them or enhance their degradation. As for nuclear translocation, the global effect is possibly mediated by regulating importins or nuclear pore proteins (NUPs), while the individual effects can be mediated by either reduced or enhanced binding to the translocation machinery or to anchoring proteins. Being such central signaling pathways, the dysregulation of the p38α/β cascade results in pathologies, and indeed, almost all constantly nuclear targets were shown to play a role in the regulation of cancer and inflammation.

One of the hallmarks of many stimulations is the rapid and robust nuclear translocation of p38α/β. This translocation is essential for the regulation of targets that are only localized in the nucleus, although it can enhance the phosphorylation of proteins that are phosphorylated in the cytoplasm and translocate to the nucleus upon stimulation (e.g., transcription factors). We found that the translocation is mediated by the binding of p38α/β with either Imp7 or Imp9, which further bind individually to Imp3. Then, the complex moves to the NUPs, where Imp3 stays, while Imp7 or Imp9 shuttles the p38α/β into the nucleus. In the nucleus, p38 is freed from the importins by Ran and then, it is able to execute its nuclear functions. The duration of p38α/β residence in the nucleus may vary between cells and conditions, after which the kinases are exported out of the nucleus by their NES-containing substrate, MK2. A few years ago, our group developed the PERY peptide that completely prevents the nuclear translocation of p38α/β and thereby prevents the growth of some cancer cells—particularly DSS-induced colon inflammation and inflammation-induced cancer. Thus, the nuclear translocation of p38α/β can serve as a good target for inflammation and cancer, and inhibitors of this kinase translocation should be further developed for clinical use.

## Abbreviations

DEF	docking site for ERK
ERK	extracellular signal-regulated kinase
Imp	importin
JNK	c-Jun N-terminal kinase
MAPK	mitogen-activated protein kinase
MAPKAPK	MAPK-activated protein kinase (also known as MK)
MKK	MAPK kinase
NUP	nuclear pore protein
